# Radiogenomic correlation of hypoxia-related biomarkers in clear cell renal cell carcinoma

**DOI:** 10.1007/s00432-025-06240-8

**Published:** 2025-06-12

**Authors:** Yijun Shao, Harmony S. Cen, Anu Dhananjay, S. J. Pawan, Xiaomeng Lei, Inderbir S. Gill, Anishka D’souza, Vinay A. Duddalwar

**Affiliations:** 1 Keck School of Medicine, University of Southern California, Los Angeles, CA USA; 2 Mark and Mary Stevens Neuroimaging and Informatics Institute, University of Southern California, Los Angeles, CA USA; 3 Newport High School, Bellevue, WA USA; 4 Radiomics Lab, Department of Radiology, Keck School of Medicine, University of Southern California, Los Angeles, CA USA; 5 Institute of Urology, University of Southern California, Los Angeles, CA USA; 6 Department of Medical Oncology, Keck School of Medicine, University of Southern California, Los Angeles, CA USA; 7 Alfred E. Mann Department of Biomedical Engineering, University of Southern California, Los Angeles, CA USA; 8 Department of Radiology, Los Angeles General Medical Center, Los Angeles, CA USA

**Keywords:** Radiomics, Clear cell renal cell carcinoma, Radiogenomics, Hypoxia, Biomarkers

## Abstract

**Purpose:**

This study aimed to evaluate radiomic models’ ability to predict hypoxia-related biomarker expression in clear cell renal cell carcinoma (ccRCC).

**Methods:**

Clinical and molecular data from 190 patients were extracted from The Cancer Genome Atlas-Kidney Renal Clear Cell Carcinoma dataset, and corresponding CT imaging data were manually segmented from The Cancer Imaging Archive. A panel of 2,824 radiomic features was analyzed, and robust, high-interscanner-reproducibility features were selected. Gene expression data for 13 hypoxia-related biomarkers were stratified by tumor grade (1/2 vs. 3/4) and stage (I/II vs. III/IV) and analyzed using Wilcoxon rank sum test. Machine learning modeling was conducted using the High-Performance Random Forest (RF) procedure in SAS Enterprise Miner 15.1, with significance at *P* < 0.05.

**Results:**

Descriptive univariate analysis revealed significantly lower expression of several biomarkers in high-grade and late-stage tumors, with KLF6 showing the most notable decrease. The RF model effectively predicted the expression of KLF6, ETS1, and BCL2, as well as PLOD2 and PPARGC1A underexpression. Stratified performance assessment showed improved predictive ability for RORA, BCL2, and KLF6 in high-grade tumors and for ETS1 across grades, with no significant performance difference across grade or stage.

**Conclusion:**

The RF model demonstrated modest but significant associations between texture metrics derived from clinical CT scans, such as GLDM and GLCM, and key hypoxia-related biomarkers including KLF6, BCL2, ETS1, and PLOD2. These findings suggest that radiomic analysis could support ccRCC risk stratification and personalized treatment planning by providing non-invasive insights into tumor biology.

**Supplementary Information:**

The online version contains supplementary material available at 10.1007/s00432-025-06240-8.

## Introduction

Renal cancer is one of the most prevalent malignant tumors worldwide (Jonasch et al. 2014). In 2024, an estimated 81,610 new cases and 14,390 deaths due to renal cancer are expected in the United States alone (Siegel et al. 2024). Approximately 90–95% of these cases are renal cell carcinoma (RCC), with clear cell RCC (ccRCC) accounting for over 70% of cases (Protzel et al. 2012). While surgical resection remains the primary treatment for localized RCC, effective therapies for metastatic RCC remain limited, with a 5-year survival rate of just 12% (Padala et al. 2020). Additionally, 20–30% of patients experience recurrence or metastasis despite nephrectomy, highlighting the need for accurate prognostic models to guide individualized treatment (Matsubara et al. 2023).

Tumor hypoxia, characterized by low partial pressures of molecular oxygen within the tumor microenvironment (TME), plays a significant role in RCC aggressiveness and treatment resistance (LaGory and Giaccia 2016; Akhtar et al. 2018). This condition arises due to a combination of excessive oxygen consumption by tumor cells and the chaotic nature of tumor-associated vasculature. Hypoxia promotes genetic instability, alters metabolic pathways, and creates an immunosuppressive TME. Hypoxia-Inducible Factor (HIF), a protein complex composed of either HIF1a or HIF2a and HIF1b/ARNT subunits, activates genes involved in angiogenesis and cell proliferation, and is central to the tumor’s adaptation to low oxygen, contributing to a neoplastic phenotype (LaGory and Giaccia 2016; Akhtar et al. 2018). As a result, hypoxia-related biomarkers have gained attention for their prognostic potential and as therapeutic targets in RCC (Zhang et al. 2021; Li et al. 2021; Gao et al. 2021).

Conventional tissue biopsy-based genetic profiling has limitations in capturing the spatial and temporal heterogeneity of tumors, leading to incomplete or inaccurate assessments (Dizman et al. 2020). Some studies have proposed the use of liquid biopsy to obtain a more complete tumor profile in a less invasive manner. However, the application of genomic profiling remains challenging in large-scale clinical practice due to limited accessibility of genetic profiling tests, expense, and genomic data validation (Pezzicoli et al. 2023). These challenges emphasize the need for scalable, non-invasive approaches to comprehensively assess tumor biology.

Radiogenomics, which correlates radiomic features from medical imaging with genomic data, presents a promising alternative. By extracting quantitative features from imaging, radiogenomics can capture tumor heterogeneity in a non-invasive and cost-effective manner. This technique utilizes automated, high-throughput feature extraction methods to analyze the biological characteristics of medical imaging data and align them with genomic information, offering valuable insights into tumor progression, development, and variability. Radiogenomics has shown significant potential in correlating non-invasive imaging features with gene expression patterns, aiding in the prediction of prognosis across various cancers, including lung and brain tumors and RCC (Mazurowski 2015; Liu et al. 2023a). In recent years, radiogenomics has expanded to include gene sequencing, expression, and tumor heterogeneity, helping guide personalized treatment strategies (Singh et al. 2021; Anagnostopoulos et al. 2022; Corr et al. 2022).

This study aims to evaluate the viability of radiomics as a surrogate marker for hypoxia-related genetic markers in ccRCC. Using The Cancer Genome Atlas-Kidney Renal Clear Cell Carcinoma (TCGA-KIRC) dataset, we applied machine learning to correlate radiomic features with the expression levels of key hypoxia-related genes. By stratifying patients by tumor stage and grade, this study seeks to enhance the predictive power of radiomic models and potentially identify imaging biomarkers for hypoxia-related gene signatures.

## Methods

### Biomarker selection

**Fig. 1:**
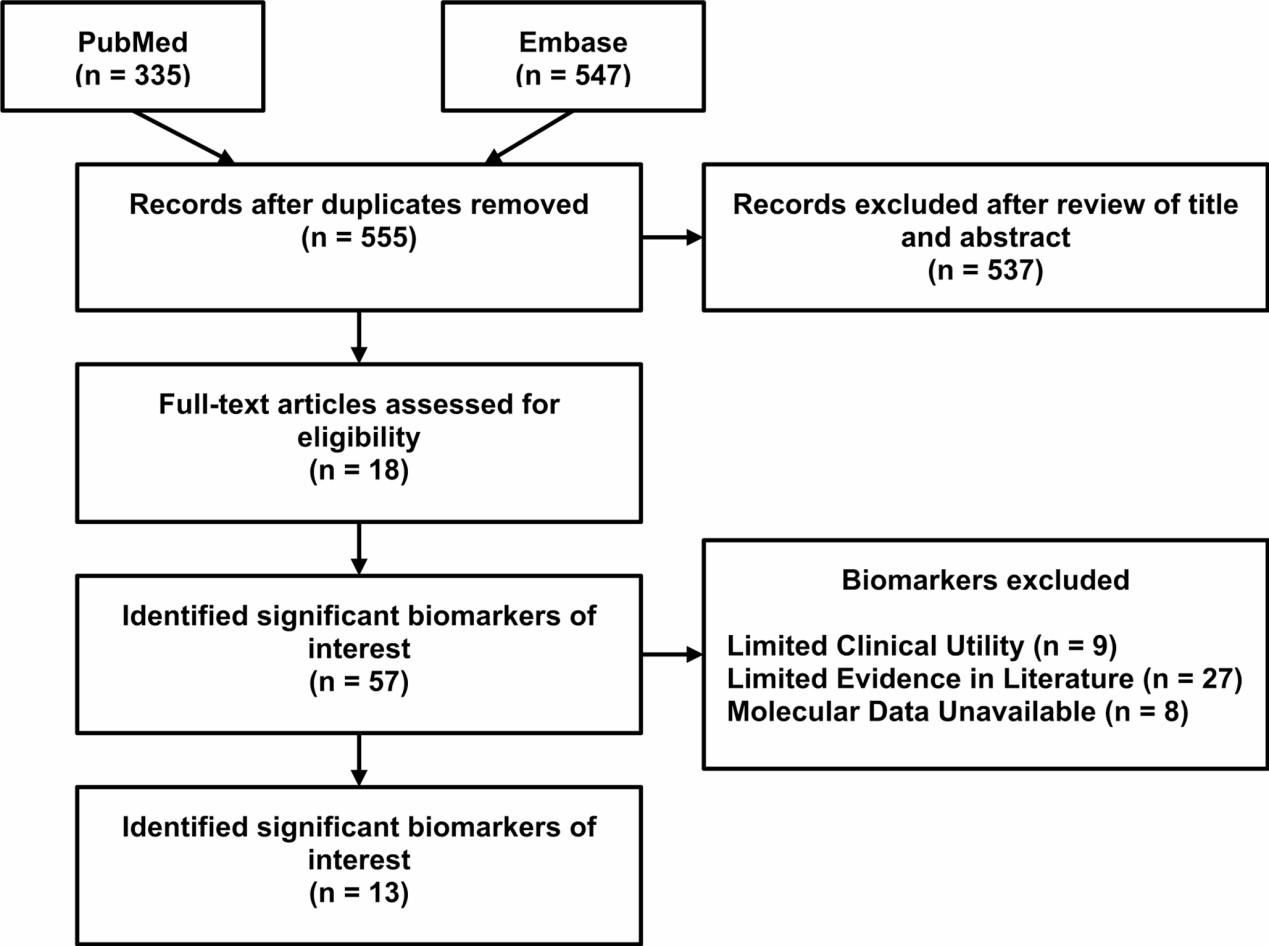
PRISMA search method from a comprehensive literature review conducted to identify hypoxia-related genetic biomarkers with significant potential for clinical prognostic and treatment response prediction

A comprehensive literature search was performed using PubMed and Embase to identify significant hypoxia-related molecular biomarkers in ccRCC (Fig. 1). Thirteen genes were selected based on (1) HIF1a or HIF2a pathway involvement, (2) prior inclusion in prognostic models or survival associations in ccRCC, and (3) availability of expression data in TCGA-KIRC (Ricketts et al. 2018). For example, KLF6 and PLOD2 have both been shown to be responsive to hypoxia and independently associated with ccRCC progression and survival (Gao et al. 2017; Liu et al. 2023b). Details of each included biomarker and its biological role are provided in Table 1.

**Table 1:** Hypoxia-Related biomarkers of interest for ccRCC

Biomarker	Description
**ANKZF1**	Ankyrin repeat and zinc finger peptidyl tRNA hydrolase 1; Cofactor involved in regulating functions including apoptosis, transcriptional activation, and DNA repair (Li et al. 2021); accumulation associated with poor overall survival (OS) (Miao et al. 2024)
**BCL2**	B-cell lymphoma 2; negative regulator of apoptosis; upregulation associated with better prognosis (Itoi et al. 2004)
**ETS1**	E26 transformation specific 1; Regulates HIF-related genes and promotes apoptosis; downregulation associated with poor prognosis (Li et al. 2021)
**FBP1**	Fructose-1,6-bisphosphatase 1; Key enzyme involved in gluconeogenesis, reduced in ccRCC; downregulation correlated with lower OS (Li et al. 2021)
**KLF6**	Kruppel-like factor 6; tumor suppressor; downregulation associated with poorer OS (Gao et al. 2017)
**PCK1**	Phosphoenolpyruvate carboxykinase 1; rate-limiting gluconeogenesis enzyme; downregulation associated with poorer prognosis (Shi et al. 2020)
**PDK1**	Key enzyme regulator of cell metabolism; upregulation associated with higher pt OS (Nunes-Xavier et al. 2023)
**PLAUR**	Receptor for urokinase plasminogen activator; promotes tumor invasion; correlated with poor prognosis (Li et al. 2021)
**PLOD2**	Procollagen-lysine, 2-oxoglutarate 5-dioxygenase 2; upregulated by HIF1a; upregulation associated with poor OS (Liu et al. 2023b)
**PPARGC1A**	Peroxisome proliferator-activated receptor gamma coactivator-1; key transcriptional coactivator coordinating metabolism; upregulation associated with improved OS (Ma et al. 2021)
**RORA**	RAR related orphan receptor A; contributes to tumor proliferation and metastasis; downregulation associated with lower OS (Huang et al. 2022)
**TEK**	Receptor Tyrosine Kinase; Controls vasculogenesis and remodeling; downregulation associated with lower OS (Ha et al. 2019)
**WSB1**	WD repeat and SOCS box-containing protein 1; regulates cell metabolism and upregulated by HIF1; upregulation associated with poorer prognosis (Tang et al. 2024)

Patients’ genomic data were sourced from the TCGA-KIRC database, while corresponding single and multiphase contrast-enhanced CT images were obtained from matched sets at The Cancer Imaging Archive (TCIA) (Clark et al. 2013). Gene expression values were normalized using the transcripts per million (TPM) method, accounting for sequencing depth and gene length. Expression levels were then binarized into high and low groups using the median value of each gene across the entire cohort. This approach aligns with prior radiogenomic studies evaluating prognostic biomarkers (Liu et al. 2023a), and was chosen to facilitate interpretable, clinically-relevant classification. While the median threshold may not always reflect a validated biological cutoff, it enables stratified analysis of imaging correlates across comparable patient subsets. All data used in this study were publicly available and fully de-identified, thereby exempting the study from Institutional Review Board (IRB) review.

### Image segmentation and radiomics analysis

Two radiology trainees independently conducted manual segmentation of renal tumor voxels under the guidance and supervision of a fellowship-trained radiologist with expertise in abdominal imaging. Tumor voxels were isolated from adjacent tissues and defined as 3D regions of interest (ROIs) using the Synapse 3D workstation (Fujifilm Medical Systems U.S.A., Stamford, CT). Any segmentation differences were resolved by reaching consensus with the supervising radiologist. Each patient had two to four available CT phases, including precontrast, corticomedullary, nephrographic, and excretory phases. For cases with multiphase imaging data available, the nephrographic phase was used as the reference point for co-registering the other phases. Most scans were acquired using GE Medical Systems scanners, followed by Siemens, with the majority of slice thickness values between 2 and 5 mm, in-plane pixel spacing around 0.55–0.9 mm, and tube voltage between 120 and 140 kV. Detailed per-case acquisition metadata is available in the DICOM headers hosted by TCIA (Clark et al. 2013). All segmentations were also reviewed by the radiologist and organized into multiphase datasets. No additional preprocessing steps, such as resizing, denoising, or normalization, were applied to the images to prevent bias and maintain data integrity (Liu et al. 2023a). Voxel data corresponding to the defined ROI were extracted using custom scripts in MATLAB^®^. 2D analysis focused on the largest tumor cross-section in each phase and imaging plane, while 3D analysis encompassed the entire tumor volume.

The radiomics panel in this study consisted of 2,824 radiomic features derived from 15 distinct texture analysis methods, extracted using a custom-built MATLAB^®^ software package (MathWorks, Natick, MA, USA). This radiomics framework, which we adopted from Varghese et al. (Varghese et al. 2021), complies with the standards set by the Image Biomarker Standardization Initiative (Yap et al. 2018; Kunapuli et al. 2018; Lei et al. 2021). A total of 388 radiomic features, identified for their high reproducibility and robustness across various imaging scanners, were selected for the robust model. Feature robustness was evaluated using intraclass correlation coefficient (ICC 3.1). 60% of all features had an ICC ≥ 0.70 across four CT scanners and were deemed robust (Varghese et al. 2021). These features spanned the following families: Gray Level Co-Occurrence Matrix (GLCM 2D/3D), Gray Level Dependence Matrix (GLDM 2D/3D), Gray Level Run Length Matrix (GLRLM 2D/3D), and Gray Level Size Zone Matrix (GLSZM 2D/3D).

### Statistical analysis and machine learning classification

Descriptive analysis of the transcriptomic expression of hypoxia-related genes, tumor grade, and stage was performed using either the t-test or Wilcoxon rank-sum test, depending on the normality of the data. A Random Forest (RF) model was employed to evaluate the predictive ability of radiomic features for the continuous transcriptomic expression of hypoxia-related genes. A dichotomized classification approach was not appropriate, as the hypoxia-related genes evaluated in this study, including several novel targets validated in vitro and in vivo, do not have established clinical cut-off values. Significance was set at *P* < 0.05. The sum of squared errors was used as the loss function for model optimization, and hyperparameter tuning was conducted to minimize this error by a 10-fold cross validation within the training phase. To address potential variable selection bias, Strobl’s method for unbiased variable selection in Random Forests was applied (Strobl et al. 2008). Model performance was evaluated using 10-fold cross-validation, which enabled a larger proportion of the data (90%) to be used for training in each iteration, while withholding 10% to prevent data leakage given the modest sample size. The final model performance assessment was conducted by correlation analysis between predicted and observed transcriptomic features. Further stratified analysis was conducted by adding an interaction term in the performance assessment model. All statistical analyses and machine learning modeling were performed using SAS Enterprise Miner 15.1, specifically leveraging the High-Performance Random Forest procedure for RF modeling.

## Results

### Patient cohort

Out of the 537 TCGA-KIRC cases, 237 were matched to corresponding CT images in TCIA. 190 of these were included because they had a tumor larger than 1 cm and had optimal image quality on evaluation by an experienced radiologist. A summary of patient characteristics is shown in Table 2 for the 190 patients. Most patients were male (65.79%), Caucasian (92.11%), and had localized disease (85.8%).

**Table 2:** Patient characteristics. (*n* = 190)

**Characteristic**	*N* (%)			*N* (%)			
	Stage I/II (*n* = 114)	Stage III/IV (*n* = 76)	*p* value	Grade 1/2 (*n* = 70)	Grade 3/4 (*n* = 120)	*p* value	Total, *N* (%)
**Age**, **median (IQR)**	58.2 (50.3–67.8)	62.6 (56.8–70.6)	0.02*	59.4 (50.9–69.8)	60.5 (52.1–69.9)	0.49	59.9 (51.9–69.9)
**Sex**							
Male	79 (69.30)	46 (60.53)	0.21	41 (58.57)	84 (70.00)	0.11	125 (65.79)
Female	35 (30.70)	30 (39.47)		29 (41.43)	36 (30.00)		65 (34.21)
**Race**							
White	107 (93.86)	68 (89.47)	0.19	66 (94.29)	109 (90.83)	0.49	175 (92.11)
Black	7 (6.14)	6 (7.89)		4 (5.71)	9 (7.50)		13 (6.84)
Asian	0 (0.00)	2 (2.63)		0 (0.00)	2 (1.67)		2 (1.05)
**Fuhrman grade**							
1	1 (0.88)	0 (0.00)	< 0.01*	1 (1.43)	--	--	1 (0.53)
2	58 (50.88)	11 (14.47)		69 (98.57)	--		69 (36.32)
3	46 (40.35)	41 (53.95)		--	87 (72.50)		87 (45.79)
4	9 (7.89)	24 (31.58)		--	33 (27.50)		33 (17.37)
**Stage**							
I	97 (85.09)	--	--	50 (71.43)	47 (39.17)	< 0.01*	97 (51.05)
II	17 (14.91)	--		9 (12.86)	8 (6.67)		17 (8.95)
III	--	45 (59.21)		10 (14.29)	35 (29.17)		45 (23.68)
IV	--	31 (40.79)		1 (1.43)	30 (25.00)		31 (16.32)

* *p* < 0.05 indicates statistical significance

### Descriptive univariate analysis

The expression levels of hypoxia-related biomarkers varied across different tumor stages and grades as shown in Table 3. In the stage-stratified analysis, BCL2, KLF6, PDK1, and PPARGC1A exhibited significantly lower expression in late-stage tumors (stage III/IV) compared to early-stage tumors (stage I/II), with *p*-values of < 0.01, < 0.01, 0.02, and < 0.01, respectively. TEK exhibited significantly higher expression in the late-stage tumors (*p* = 0.03). In the grade-stratified analysis, ETS1, KLF6, PDK1, and TEK showed significantly reduced expression in high grade tumors (grades 3/4), with *p*-values of 0.01, < 0.01, 0.04, and 0.01, respectively. Among these biomarkers, KLF6 demonstrated the most pronounced reduction in expression with tumor progression, as evidenced by significant decreases in both stage and grade-stratified analyses.

**Table 3:** Median and interquartile range of TPM-Normalized gene expression of specified biomarkers, unstratified and stratified by grade (1/2 vs. 3/4) and stage (I/II vs. III/IV)

Biomarker	Median (IQR)			Median (IQR)			
	Stage I/II (*n* = 114)	Stage III/IV (*n* = 76)	*p* value	Grade 1/2 (*n* = 70)	Grade 3/4 (*n* = 120)	*p* value	Unstratified (*n* = 190)
**ANKZF1**	17.87 (10.46–25.38)	21.19 (10.83–32.07)	0.32	16.85 (10.03–25.10)	19.13 (11.02–31.03)	0.22	18.48 (10.66–29.74)
**BCL2**	26.41 (13.85–42.96)	17.73 (11.01–31.51)	< 0.01*	26.72 (15.54–37.90)	20.98 (12.88–36.72)	0.10	23.99 (13.06–37.53)
**ETS1**	138.71 (70.75-220.67)	112.96 (66.67-173.57)	0.08	160.90 (84.51-238.69)	119.16 (64.53-177.77)	0.01*	131.07 (67.47-200.88)
**FBP1**	39.63 (16.19–66.66)	26.16 (12.18–50.95)	0.13	37.44 (17.64–60.35)	34.74 (12.70-59.39)	0.46	36.10 (13.07-60.00)
**KLF6**	214.39 (123.57-343.52)	151.85 (90.09–236.10)	< 0.01*	245.56 (131.24-364.72)	155.61 (92.06-251.72)	< 0.01*	182.11 (108.36-291.49)
**PCK1**	40.69 (9.09-100.63)	19.44 (3.64–97.75)	0.14	39.84 (8.28–95.28)	28.78 (6.29–99.82)	0.49	32.13 (6.52–99.49)
**PDK1**	14.27 (7.66–19.71)	10.64 (6.18–16.29)	0.02*	14.87 (9.47–21.62)	12.44 (6.56–17.97)	0.04*	13.38 (6.91–18.88)
**PLAUR**	11.00 (5.79–17.84)	12.50 (6.23–18.67)	0.39	9.29 (5.89–15.17)	12.73 (5.89–20.65)	0.08	11.54 (5.87–18.57)
**PLOD2**	92.73 (59.57-145.36)	89.84 (46.94-149.04)	0.76	89.88 (55.19-154.36)	93.20 (54.57-145.84)	0.68	91.20 (54.75-146.89)
**PPARGC1A**	11.01 (5.63–17.37)	6.48 (3.20-12.46)	< 0.01*	10.49 (5.63–15.76)	7.50 (3.98–16.99)	0.37	8.14 (4.70-16.02)
**RORA**	9.31 (6.16–14.86)	8.04 (5.24–10.94)	0.04*	9.37 (5.78–14.86)	8.56 (5.55–12.88)	0.25	8.75 (5.56–13.28)
**TEK**	28.13 (15.33–50.85)	29.76 (10.32–36.04)	0.03*	31.31 (18.11–55.23)	23.76 (10.32–37.60)	0.01*	25.53 (11.34–45.85)
**WSB1**	32.03 (22.80-61.85)	40.05 (23.49–66.91)	0.51	32.93 (22.47–70.05)	35.08 (24.48–62.42)	0.86	34.42 (22.94–64.05)

* *p* < 0.05 indicates statistical significance

### RF robust model performance analysis

The RF model trained on robust features showed varying predictive performance across hypoxia-related biomarkers (Online Resources 1–3). In the unstratified analysis, the model showed notable predictive power for the expression of KLF6 (*r* = 0.27 (0.14–0.41), *p* < 0.01), ETS1 (*r* = 0.25 (0.11–0.39), *p* < 0.01), and BCL2 (*r* = 0.19 (0.05–0.33), *p* < 0.01), as well as an underexpression of PLOD2 (*r* = -0.19 (-0.33–-0.05), *p* < 0.01) and PPARGC1A (*r* = -0.15 (-0.29–0.01), *p* = 0.04). All confidence intervals are at 95%.

Stratified results demonstrated higher predictive ability for the expression of RORA (*r* = 0.25 (0.04–0.46), *p* = 0.02), BCL2 (*r* = 0.27 (0.09–0.44), *p* < 0.01), and KLF6 (*r* = 0.35 (0.14–0.56), *p* < 0.01) in high grade, as well as ETS1 in both grades (high: *r* = 0.20 (0.01–0.40), *p* = 0.04; low: *r* = 0.23 (0.03–0.43), *p* = 0.02), and underexpression of PLOD2 in high grade (*r* = -0.23 (-0.39–0.06), *p* < 0.01).

For stage stratification, underexpression of PLOD2 was better predicted in high stage (*r* = -0.22 (-0.41–-0.03), *p* = 0.02), while the expression of BCL2 (*r* = 0.20 (0.02–0.38), *p* = 0.03), ETS1 (*r* = 0.24 (0.08–0.41), *p* < 0.01), and KLF6 (*r* = 0.22 (0.06–0.38), *p* < 0.01) was better predicted in low stage. In both stratified analyses, there was no statistically significant difference between high/low grade and stage, indicating similar performance across these stratifications. Results for the RF model trained on all radiomic features are in Online Resources 4–6.

### Radiomic features of importance

**Fig. 2:**
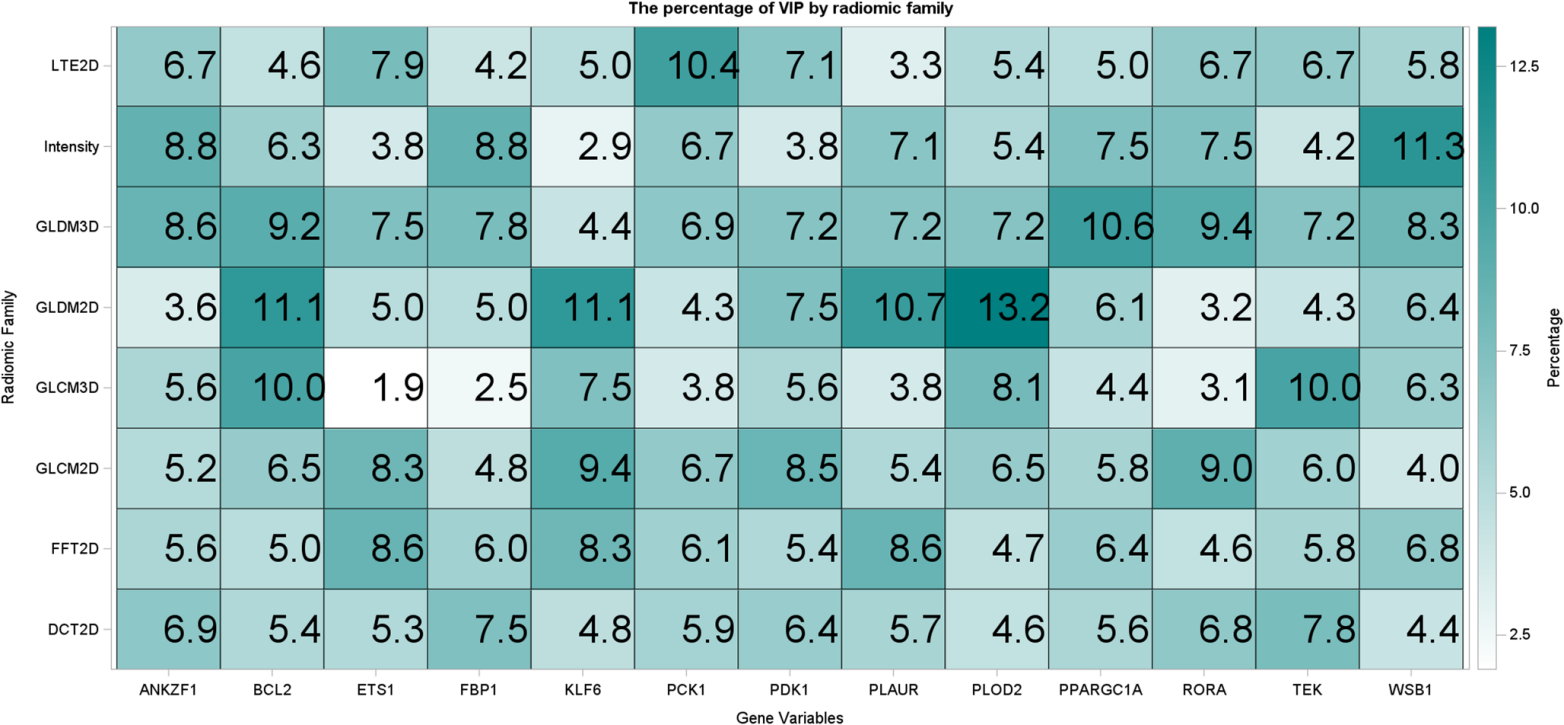
Heatmap illustrating the distribution of robust radiomic features identified as important predictors in the machine learning model across hypoxia-related genes. Each cell represents the percentage of features from a given texture family contributing to the prediction of a specific gene. Darker shades indicate a higher proportion of important features, with values ranging from 0% (white) to approximately 14% (teal blue)

Preliminary analyses identified the most critical features for each molecular marker within the RF robust model. Texture methods were evaluated based on the percentage of features classified as variables of importance (VOI). Several biomarkers, including BCL2, KLF6, PLOD2, and PPARGC1A, showed acceptable discrimination with over 10% of features classified as VOI in some methods. Notably, GLDM 2D classified over 10% of features predictive of BCL2, KLF6, and PLOD2 and GLCM 3D of BCL2 and KLF6 (see Fig. 2). Feature families are defined in Table 4. Top individual features predictive for each gene are summarized in Online Resource 7. While certain features, such as GD2_Cor_DEL_Uniformity, were important across multiple genes (BCL2, RORA), most genes exhibited distinct top features, suggesting gene-specific radiomic signatures. Feature definitions and abbreviations are provided in Online Resource 8.

**Table 4:** Radiomic texture methods included in the robust model

Radiomic Method	Abbreviation	Description
LTE 2D	LE2	Laws’ texture energy measures − 2D
INT	INT	Histogram analysis − 3D
GLDM 3D or 2D	GD3 or GD2	Gray level difference matrix − 3D or 2D
GLCM 3D or 2D	GC3 or GC2	Gray level co-occurrence matrix − 3D or 2D
GLRLM 3D or 2D	LR3 or LR2	Gray level run length matrix − 3D or 2D
GLSZM 3D or 2D	LS3 or LS2	Gray level size zone matrix − 3D or 2D
FFT 2D	FT2	Fast Fourier transform − 2D
DCT 2D	DT2	Discrete cosine transform − 2D

## Discussion

This study evaluated radiomic features derived from standard-of-care clinical CT scans as potential non-invasive correlates of hypoxia-related biomarker expression in ccRCC. Using our RF machine learning model trained on robust texture features, we identified statistically significant but modest associations between radiomic patterns and the expression of select hypoxia-related genes, particularly KLF6, BCL2, and ETS1. Stratified analyses by tumor grade showed moderate improvements in predictive performance, suggesting that underlying tumor biology may influence the strength of radiogenomic associations.

The RF model demonstrated significant predictive performance for specific biomarkers, most notably KLF6, BCL2, and ETS1. Heatmap analysis (shown in Fig. 2) highlights GLDM 2D and GLCM 3D features as substantial contributors to predictive accuracy for KLF6 and BCL2. GLDM features measure pixel dependencies, capturing regions with consistent texture that may correspond to structural changes such as necrosis or hypoxia-induced tissue remodeling, common in aggressive ccRCC tumors. GLCM features, which quantify spatial relationships between pixel intensities, reflect patterns of intensity variation that may correlate with cellular density and spatial heterogeneity within the tumor. These texture metrics provide valuable insights into the heterogeneous tumor microenvironment typical of advanced ccRCC. KLF6’s downregulation in high-grade tumors aligns with its tumor suppressor role and association with poorer outcomes (Gao et al. 2017). Similarly, BCL2, an apoptosis regulator linked to better prognosis due to their association with tumors of slower growth and better-organized tissue architecture, was effectively predicted by GLDM and GLCM features, supporting the utility of texture-based radiomics in capturing hypoxia-related alterations​ (Itoi et al. 2004). Additionally, BCL2 was better predicted in our stratified analysis at the high-grade, low-stage groups, characteristic of localized aggressive tumors prior to metastasis, further demonstrating its potential as an early therapeutic target for intervention before disease progression.

Interestingly, although ETS1 expression prediction did not rely heavily on specific radiomic features in the heatmap, it still emerged as significantly predicted in both the unstratified and stratified models. ETS1, a transcription factor involved in HIF-related pathways, likely reflects tumor-wide adaptations to hypoxia that manifest as a broader radiomic signature rather than being linked to specific texture patterns (Li et al. 2021). This suggests that, while ETS1’s expression may not correlate strongly with individual radiomic features, its significance in the overall model underscores the complex, diffuse nature of hypoxia-related adaptations in ccRCC.

PLOD2 showed unique behavior, with a negative correlation coefficient, particularly in high-grade, late-stage tumors. This inverse relationship suggests that higher PLOD2 expression corresponds to lower radiomic texture feature values. GLDM 2D was the most predictive feature for PLOD2, potentially capturing areas within the tumor where structural remodeling leads to a reduction in consistent texture—a phenomenon likely related to hypoxia-induced collagen cross-linking and stiffening (Liu et al. 2023b). PLOD2’s role in collagen formation and extracellular matrix modification under hypoxic conditions is a possible explanation, making it an interesting biomarker for further research.

These findings support radiomic features as potential non-invasive predictors of hypoxia-related gene expression, aiding in risk stratification and guiding treatment decisions. Specifically, KLF6, BCL2, ETS1, and PLOD2 could provide insights into tumor biology, hypoxia adaptations, and aggressiveness without invasive biopsies. The significance of texture metrics like GLDM and GLCM in our model suggests they can act as proxies for the spatial organization and heterogeneity within ccRCC lesions.

To further explore the specific radiomic patterns underlying these associations, we analyzed individual features most predictive for each gene. Beyond family-level trends, specific radiomic features emerged as predictors for certain genes. For instance, GD2_Axi_VEN_HOM and DT2_Axi_DEL_skew_dB_4 were highly important for KLF6, aligning with the role of hypoxia in disrupting tissue homogeneity. For BCL2, features such as GD2_Cor_DEL_Uniformity, LR2_Sag_PRE_LGRE, and GD2_Axi_DEL_difENT were among the top predictors, reflecting patterns of preserved uniformity, low gray-level emphasis, and differences in entropy, respectively. These findings are consistent with BCL2’s association with slow growth and increased tissue organization. Features capturing entropy, uniformity, and skewness were consistently among the most predictive, supporting their biological relevance to hypoxia-induced tumor architecture changes.

This study has several limitations. First, the sample size, restricted to high-quality imaging and complete genomic data from TCGA-KIRC, limits generalizability, compounded by a predominantly Caucasian and male cohort. In addition, the use of a single institutional cohort without external validation limits the generalizability of the findings. Future work should focus on independent validation to strengthen these results. Second, using whole-tumor imaging without precise biopsy locations may cause discrepancies when correlating radiomic features with gene expression, as biopsies might not align with specific tumor regions captured in scans. Additionally, reliance on CT-based radiomics could be enhanced with multimodal imaging like MRI or PET for more comprehensive tumor data. The abstract nature of radiomic features poses challenges for interpretation; integrating them with functional imaging or spatial transcriptomics could help improve interpretability. Additionally, the cross-sectional nature of our data limits tracking of radiomic changes over time, which could be addressed with longitudinal imaging. Finally, while the RF model demonstrated satisfactory performance, the application of deep learning algorithms on larger datasets could further enhance model robustness and mitigate overfitting.

In conclusion, this study demonstrates the potential of radiomic features to serve as non-invasive correlates of hypoxia-related gene expression in ccRCC, particularly in advanced tumors where hypoxia plays a greater role. These findings suggest that radiogenomic analysis could, with further validation, be integrated into clinical workflows as a non-invasive tool for risk stratification. By leveraging imaging data to predict hypoxia-related biomarker expression, radiomics may eventually enable clinicians to assess tumor aggressiveness and inform personalized treatment strategies without relying solely on invasive procedures.

## Electronic supplementary material

Below is the link to the electronic supplementary material.

Supplementary Material 1

Supplementary Material 2

Supplementary Material 3

Supplementary Material 4

Supplementary Material 5

Supplementary Material 6

Supplementary Material 7

Supplementary Material 8

## Data Availability

Publicly available datasets were used in this study, found in The Cancer Genome Atlas-Kidney Renal Clear Cell Carcinoma (TCGA-KIRC) at http://doi.org/10.1016/j.celrep.2018.03.075 (Ricketts et al. 2018). Further inquiries can be directed to the corresponding author.

## References

[CR1] Akhtar M, Al-Bozom IA, Al Hussain T (2018) Molecular and metabolic basis of clear cell carcinoma of the kidney. Adv Anat Pathol 25:189–196. 10.1097/PAP.0000000000000185 29465421 10.1097/PAP.0000000000000185

[CR2] Anagnostopoulos AK, Gaitanis A, Gkiozos I et al (2022) Radiomics/Radiogenomics in lung cancer: basic principles and initial clinical results. Cancers 14:1657. 10.3390/cancers14071657 35406429 10.3390/cancers14071657 PMC8997041

[CR3] Clark K, Vendt B, Smith K et al (2013) The Cancer imaging archive (TCIA): maintaining and operating a public information repository. J Digit Imaging 26:1045–1057. 10.1007/s10278-013-9622-7 23884657 10.1007/s10278-013-9622-7 PMC3824915

[CR4] Corr F, Grimm D, Saß B et al (2022) Radiogenomic predictors of recurrence in Glioblastoma—A systematic review. J Pers Med 12:402. 10.3390/jpm12030402 35330402 10.3390/jpm12030402 PMC8952807

[CR5] Dizman N, Philip EJ, Pal SK (2020) Genomic profiling in renal cell carcinoma. Nat Rev Nephrol 16:435–451. 10.1038/s41581-020-0301-x 32561872 10.1038/s41581-020-0301-x

[CR7] Gao Y, Li H, Ma X et al (2017) KLF6 suppresses metastasis of clear cell renal cell carcinoma via transcriptional repression of E2F1. Cancer Res 77:330–342. 10.1158/0008-5472.CAN-16-0348 27780824 10.1158/0008-5472.CAN-16-0348

[CR6] Gao J, Ye F, Han F et al (2021) A novel radiogenomics biomarker based on Hypoxic-Gene subset: accurate survival and prognostic prediction of renal clear cell carcinoma. 10.3389/fonc.2021.739815. Front Oncol 11:10.3389/fonc.2021.739815 PMC8529272 34692518

[CR8] Ha M, Son YR, Kim J et al (2019) TEK is a novel prognostic marker for clear cell renal cell carcinoma. Eur Rev Med Pharmacol Sci 23:1451–1458. 10.26355/eurrev_201902_17102 30840266 10.26355/eurrev_201902_17102

[CR9] Huang Z, Kang W, Zhang Q (2022) N6-methyladenosine methylation related immune biomarkers correlates with clinicopathological characteristics and prognosis in clear cell renal cell carcinoma. Transl Cancer Res 11:1576–1586. 10.21037/tcr-21-1953 35836532 10.21037/tcr-21-1953 PMC9273710

[CR10] Itoi T, Yamana K, Bilim V et al (2004) Impact of frequent Bcl-2 expression on better prognosis in renal cell carcinoma patients. Br J Cancer 90:200. 10.1038/sj.bjc.6601454 14710230 10.1038/sj.bjc.6601454 PMC2395310

[CR11] Jonasch E, Gao J, Rathmell WK (2014) Renal cell carcinoma. BMJ 349:g4797. 10.1136/bmj.g4797 25385470 10.1136/bmj.g4797 PMC4707715

[CR12] Kunapuli G, Varghese BA, Ganapathy P et al (2018) A Decision-Support tool for renal mass classification. J Digit Imaging 31:929–939. 10.1007/s10278-018-0100-0 29980960 10.1007/s10278-018-0100-0 PMC6261185

[CR13] LaGory EL, Giaccia AJ (2016) The ever expanding role of HIF in tumour and stromal biology. Nat Cell Biol 18:356–365. 10.1038/ncb3330 27027486 10.1038/ncb3330 PMC4898054

[CR14] Lei M, Varghese B, Hwang D et al (2021) Benchmarking various radiomic toolkit features while applying the image biomarker standardization initiative toward clinical translation of radiomic analysis. J Digit Imaging 34:1156–1170. 10.1007/s10278-021-00506-6 34545475 10.1007/s10278-021-00506-6 PMC8554949

[CR15] Li Z, Du G, Zhao R et al (2021) Identification and validation of a hypoxia-related prognostic signature in clear cell renal cell carcinoma patients. Med (Baltim) 100:e27374. 10.1097/MD.0000000000027374 10.1097/MD.0000000000027374 PMC8483867 34596153

[CR16] Liu DH, Dani KA, Reddy SS et al (2023a) Radiogenomic associations clear cell renal cell carcinoma: an exploratory study. Oncology 101:375–388. 10.1159/000530719 37080171 10.1159/000530719

[CR17] Liu T, Xiang W, Chen Z et al (2023b) Hypoxia-induced PLOD2 promotes clear cell renal cell carcinoma progression via modulating EGFR-dependent AKT pathway activation. Cell Death Dis 14:774. 10.1038/s41419-023-06298-7 38008826 10.1038/s41419-023-06298-7 PMC10679098

[CR18] Ma T, Meng L, Wang X et al (2021) TNFSF13B and PPARGC1A expression is associated with tumor-infiltrating immune cell abundance and prognosis in clear cell renal cell carcinoma. Am J Transl Res 13:11048–11064 34786042 PMC8581857

[CR19] Matsubara S, Saito A, Tokuyama N et al (2023) Recurrence prediction in clear cell renal cell carcinoma using machine learning of quantitative nuclear features. Sci Rep 13:11035. 10.1038/s41598-023-38097-7 37419897 10.1038/s41598-023-38097-7 PMC10328910

[CR20] Mazurowski MA (2015) Radiogenomics: what it is and why it is important. J Am Coll Radiol 12:862–866. 10.1016/j.jacr.2015.04.019 26250979 10.1016/j.jacr.2015.04.019

[CR21] Miao D, Shi J, Lv Q et al (2024) NAT10-mediated ac4C‐modified ANKZF1 promotes tumor progression and lymphangiogenesis in clear‐cell renal cell carcinoma by attenuating YWHAE‐driven cytoplasmic retention of YAP1. Cancer Commun 44:361. 10.1002/cac2.12523 10.1002/cac2.12523 PMC10962679 38407929

[CR22] Nunes-Xavier CE, Emaldi M, Mingo J et al (2023) The expression pattern of pyruvate dehydrogenase kinases predicts prognosis and correlates with immune exhaustion in clear cell renal cell carcinoma. Sci Rep 13:7339. 10.1038/s41598-023-34087-x 37147361 10.1038/s41598-023-34087-x PMC10162970

[CR23] Padala SA, Barsouk A, Thandra KC et al (2020) Epidemiology of renal cell carcinoma. World J Oncol 11:79–87. 10.14740/wjon1279 32494314 10.14740/wjon1279 PMC7239575

[CR24] Pezzicoli G, Ciciriello F, Musci V et al (2023) Genomic profiling and molecular characterization of clear cell renal cell carcinoma. Curr Oncol 30:9276–9290. 10.3390/curroncol30100670 37887570 10.3390/curroncol30100670 PMC10605358

[CR25] Protzel C, Maruschke M, Hakenberg OW (2012) Epidemiology, aetiology, and pathogenesis of renal cell carcinoma. Eur Urol Suppl 11:52–59. 10.1016/j.eursup.2012.05.002

[CR26] Ricketts CJ, De Cubas AA, Fan H et al (2018) The Cancer genome atlas comprehensive molecular characterization of renal cell carcinoma. Cell Rep 23:313–326e5. 10.1016/j.celrep.2018.03.075 29617669 10.1016/j.celrep.2018.03.075 PMC6075733

[CR27] Shi L, An S, Liu Y et al (2020) PCK1 regulates Glycolysis and tumor progression in clear cell renal cell carcinoma through LDHA. OncoTargets Ther 13:2613–2627. 10.2147/OTT.S241717 10.2147/OTT.S241717 PMC7125947 32280238

[CR28] Siegel RL, Giaquinto AN, Jemal A (2024) Cancer statistics, 2024. CA Cancer J Clin 74:12–49. 10.3322/caac.21820 38230766 10.3322/caac.21820

[CR29] Singh G, Manjila S, Sakla N et al (2021) Radiomics and radiogenomics in gliomas: a contemporary update. Br J Cancer 125:641–657. 10.1038/s41416-021-01387-w 33958734 10.1038/s41416-021-01387-w PMC8405677

[CR30] Strobl C, Boulesteix A-L, Kneib T et al (2008) Conditional variable importance for random forests. BMC Bioinformatics 9:307. 10.1186/1471-2105-9-307 18620558 10.1186/1471-2105-9-307 PMC2491635

[CR31] Tang G, Liu J, Gao X et al (2024) circWSB1 promotes tumor progression in CcRCC via circWSB1/miR-182-5p/WSB1 axis. Int J Biol Macromol 256:128338. 10.1016/j.ijbiomac.2023.128338 38007007 10.1016/j.ijbiomac.2023.128338

[CR32] Varghese BA, Hwang D, Cen SY et al (2021) Identification of robust and reproducible CT-texture metrics using a customized 3D-printed texture Phantom. J Appl Clin Med Phys 22:98–107. 10.1002/acm2.13162 33434374 10.1002/acm2.13162 PMC7882093

[CR33] Yap FY, Hwang DH, Cen SY et al (2018) Quantitative contour analysis as an Image-based discriminator between benign and malignant renal tumors. Urology 114:121–127. 10.1016/j.urology.2017.12.018 29305199 10.1016/j.urology.2017.12.018

[CR34] Zhang Z, Li Q, Wang F et al (2021) Identifying hypoxia characteristics to stratify prognosis and assess the tumor immune microenvironment in renal cell carcinoma. Front Genet 12. 10.3389/fgene.2021.606816 10.3389/fgene.2021.606816 PMC8238406 34194463

