# Supplementary material for: Radiogenomic correlation of hypoxia-related biomarkers in clear cell renal cell carcinoma
Source: J Cancer Res Clin Oncol. 2025 Jun 12;151(6):186. doi: 10.1007/s00432-025-06240-8 (PMC12159112; doi:10.1007/s00432-025-06240-8)
Supplement: Supplementary file 1 — Supplementary Material 1 [file 432_2025_6240_MOESM1_ESM.pdf]

Article Title: Hypoxia-Related Gene Expression in Renal Cell Carcinoma
Journal Name: Clinical and Translational Oncology
Authors: Yijun Shao, Harmony S. Cen, Anu Dhananjay, S. J. Pawan, Xiaomeng Lei, Inderbir S. Gill, Anishka D'souza, Vinay A. Duddalwar
Corresponding Author: Yijun Shao (yijunsha@usc.edu)
Affiliation: Keck School of Medicine, University of Southern California, Los Angeles, CA, USA

Online Resource 1. Random Forest (RF) Performance of Using Robust Radiomic Features to Predict TPM-normalized Gene Expression

	**Unstratified**	
**Biomarker**	**Correlation Coefficient (95%CI)**	**p value**
**ANKZF1**	-0.06 (-0.2, 0.09)	0.44
**BCL2**	0.19 (0.05, 0.33)	<0.01*
**ETS1**	0.25 (0.11, 0.39)	<0.01*
**FBP1**	0.01 (-0.13, 0.16)	86
**KLF6**	0.27 (0.14, 0.41)	<0.01*
**PCK1**	-0.01 (-0.15, 0.14)	0.93
**PDK1**	0.13 (-0.01, 0.27)	0.07
**PLAUR**	0.04 (-0.1, 0.18)	0.57
**PLOD2**	-0.19 (-0.33, -0.05)	<0.01*
**PPARGC1A**	-0.15 (-0.29, -0.01)	0.04*
**RORA**	0.12 (-0.02, 0.26)	0.09
**TEK**	0.04 (-0.1, 0.18)	0.58
**WSB1**	-0.13 (-0.27, 0.01)	0.07

* $p < 0.05$ indicates statistical significance.
