# Supplementary material for: Radiogenomic correlation of hypoxia-related biomarkers in clear cell renal cell carcinoma
Source: J Cancer Res Clin Oncol. 2025 Jun 12;151(6):186. doi: 10.1007/s00432-025-06240-8 (PMC12159112; doi:10.1007/s00432-025-06240-8)
Supplement: Supplementary file 2 — Supplementary Material 2 [file 432_2025_6240_MOESM2_ESM.pdf]

Article Title: Hypoxia-Related Gene Expression in Renal Cell Carcinoma
Journal Name: Clinical and Translational Oncology
Authors: Yijun Shao, Harmony S. Cen, Anu Dhananjay, S. J. Pawan, Xiaomeng Lei, Inderbir S. Gill, Anishka D'souza, Vinay A. Duddalwar
Corresponding Author: Yijun Shao (yijunsha@usc.edu)
Affiliation: Keck School of Medicine, University of Southern California, Los Angeles, CA, USA

Online Resource 2. Random Forest (RF) Performance Stratified by Grade Based on Robust Radiomic Features Only

Biomarker	Grade 1/2		Grade 3/4		Difference	
	Correlation Coefficient (95%CI)	p value	Correlation Coefficient (95%CI)	p value	Correlation Coefficient (95%CI)	p value
**ANKZF1**	-0.13 (-0.37, 0.12)	0.31	-0.03 (-0.21, 0.15)	0.75	0.1 (-0.2, 0.4)	0.52
**BCL2**	0.03 (-0.2, 0.27)	0.8	0.27 (0.09, 0.44)	<0.01*	0.24 (-0.06, 0.53)	0.11
**ETS1**	0.23 (0.03, 0.43)	0.02*	0.2 (0.01, 0.4)	0.04*	-0.03 (-0.31, 0.25)	0.85
**FBP1**	0.04 (-0.33, 0.4)	0.84	0.01 (-0.14, 0.17)	0.87	-0.02 (-0.42, 0.37)	0.91
**KLF6**	0.13 (-0.06, 0.32)	0.18	0.35 (0.14, 0.56)	<0.01*	0.22 (-0.06, 0.5)	0.12
**PCK1**	-0.1 (-0.35, 0.15)	0.44	0.04 (-0.14, 0.21)	0.67	0.14 (-0.17, 0.44)	0.38
**PDK1**	0.06 (-0.15, 0.27)	0.57	0.19 (-0.01, 0.38)	0.06	0.13 (-0.16, 0.42)	0.39
**PLAUR**	-0.24 (-0.6, 0.12)	0.2	0.08 (-0.08, 0.23)	0.32	0.32 (-0.08, 0.71)	0.12
**PLOD2**	-0.1 (-0.37, 0.17)	0.48	-0.23 (-0.39, -0.06)	<0.01*	-0.13 (-0.45, 0.19)	0.42
**PPARGC1A**	-0.2 (-0.46, 0.06)	0.13	-0.12 (-0.29, 0.05)	0.17	0.08 (-0.23, 0.39)	0.61
**RORA**	-0.02 (-0.21, 0.17)	0.86	0.25 (0.04, 0.46)	0.02*	0.27 (-0.01, 0.55)	0.07
**TEK**	0.05 (-0.18, 0.27)	0.68	-0.02 (-0.21, 0.16)	0.81	-0.07 (-0.36, 0.22)	0.63
**WSB1**	-0.16 (-0.41, 0.09)	0.22	-0.12 (-0.29, 0.05)	0.17	0.04 (-0.27, 0.34)	0.82

* $p < 0.05$ indicates statistical significance.
