# Supplementary material for: Radiogenomic correlation of hypoxia-related biomarkers in clear cell renal cell carcinoma
Source: J Cancer Res Clin Oncol. 2025 Jun 12;151(6):186. doi: 10.1007/s00432-025-06240-8 (PMC12159112; doi:10.1007/s00432-025-06240-8)
Supplement: Supplementary file 3 — Supplementary Material 3 [file 432_2025_6240_MOESM3_ESM.pdf]

Article Title: Hypoxia-Related Gene Expression in Renal Cell Carcinoma
Journal Name: Clinical and Translational Oncology
Authors: Yijun Shao, Harmony S. Cen, Anu Dhananjay, S. J. Pawan, Xiaomeng Lei, Inderbir S. Gill, Anishka D'souza, Vinay A. Duddalwar
Corresponding Author: Yijun Shao (yijunsha@usc.edu)
Affiliation: Keck School of Medicine, University of Southern California, Los Angeles, CA, USA

Online Resource 3. Random Forest (RF) Performance Stratified by Stage Based on Robust Radiomic Features Only

Biomarker	Stage I/II		Stage III/IV		Difference	
	Correlation Coefficient (95%CI)	p value	Correlation Coefficient (95%CI)	p value	Correlation Coefficient (95%CI)	p value
**ANKZF1**	-0.05 (-0.24, 0.15)	0.62	-0.07 (-0.29, 0.14)	0.52	-0.02 (-0.31, 0.27)	0.88
**BCL2**	0.2 (0.02, 0.38)	0.03*	0.1 (-0.13, 0.33)	0.41	-0.1 (-0.39, 0.19)	0.51
**ETS1**	0.24 (0.08, 0.41)	<0.01*	0.14 (-0.11, 0.39)	0.26	-0.1 (-0.4, 0.2)	0.51
**FBP1**	0.02 (-0.16, 0.21)	0.79	0 (-0.24, 0.23)	0.99	-0.03 (-0.32, 0.27)	0.87
**KLF6**	0.22 (0.06, 0.38)	<0.01*	0.19 (-0.08, 0.46)	0.17	-0.03 (-0.35, 0.28)	0.84
**PCK1**	-0.01 (-0.17, 0.14)	0.86	0.03 (-0.35, 0.4)	0.88	0.04 (-0.36, 0.45)	0.83
**PDK1**	0.03 (-0.14, 0.2)	0.72	0.21 (-0.03, 0.46)	0.09	0.18 (-0.12, 0.48)	0.23
**PLAUR**	-0.15 (-0.47, 0.16)	0.33	0.06 (-0.1, 0.22)	0.45	0.22 (-0.14, 0.57)	0.23
**PLOD2**	-0.15 (-0.37, 0.06)	0.16	-0.22 (-0.41, -0.03)	0.02*	-0.07 (-0.35, 0.22)	0.65
**PPARGC1A**	-0.13 (-0.3, 0.03)	0.12	-0.21 (-0.5, 0.07)	0.15	-0.08 (-0.41, 0.25)	0.63
**RORA**	0.05 (-0.11, 0.21)	0.54	0.19 (-0.12, 0.5)	0.23	0.14 (-0.2, 0.49)	0.42
**TEK**	0.01 (-0.17, 0.18)	0.94	0.01 (-0.24, 0.26)	0.92	0.01 (-0.3, 0.31)	0.97
**WSB1**	-0.12 (-0.29, 0.06)	0.19	-0.16 (-0.41, 0.09)	0.2	-0.05 (-0.35, 0.26)	0.76

* $p < 0.05$ indicates statistical significance.
