# Supplementary material for: Radiogenomic correlation of hypoxia-related biomarkers in clear cell renal cell carcinoma
Source: J Cancer Res Clin Oncol. 2025 Jun 12;151(6):186. doi: 10.1007/s00432-025-06240-8 (PMC12159112; doi:10.1007/s00432-025-06240-8)
Supplement: Supplementary file 4 — Supplementary Material 4 [file 432_2025_6240_MOESM4_ESM.pdf]

Article Title: Hypoxia-Related Gene Expression in Renal Cell Carcinoma
Journal Name: Clinical and Translational Oncology
Authors: Yijun Shao, Harmony S. Cen, Anu Dhananjay, S. J. Pawan, Xiaomeng Lei, Inderbir S. Gill, Anishka D'souza, Vinay A. Duddalwar
Corresponding Author: Yijun Shao (yijunsha@usc.edu)
Affiliation: Keck School of Medicine, University of Southern California, Los Angeles, CA, USA

Online Resource 4. Random Forest (RF) Performance of Using Radiomic Features to Predict TPM-normalized Gene Expression Based on All Available Radiomic Features

Biomarker	Unstratified	
	Correlation Coefficient (95%CI)	p value
ANKZF1	-0.04 (-0.18, 0.11)	0.62
BCL2	0.05 (-0.09, 0.19)	0.5
ETS1	0.14 (0, 0.28)	0.05*
FBP1	0 (-0.14, 0.15)	0.96
KLF6	0.2 (0.06, 0.34)	<0.01*
PCK1	0.04 (-0.1, 0.19)	0.56
PDK1	0.06 (-0.09, 0.2)	0.43
PLAUR	0.01 (-0.13, 0.15)	0.87
PLOD2	-0.08 (-0.23, 0.06)	0.26
PPARGC1A	-0.09 (-0.24, 0.05)	0.2
RORA	0.07 (-0.08, 0.21)	0.36
TEK	-0.07 (-0.21, 0.07)	0.33
WSB1	-0.06 (-0.2, 0.09)	0.44

* $p < 0.05$ indicates statistical significance.
