# Supplementary material for: Radiogenomic correlation of hypoxia-related biomarkers in clear cell renal cell carcinoma
Source: J Cancer Res Clin Oncol. 2025 Jun 12;151(6):186. doi: 10.1007/s00432-025-06240-8 (PMC12159112; doi:10.1007/s00432-025-06240-8)
Supplement: Supplementary file 5 — Supplementary Material 5 [file 432_2025_6240_MOESM5_ESM.pdf]

**Article Title:** Hypoxia-Related Gene Expression in Renal Cell Carcinoma
**Journal Name:** Clinical and Translational Oncology
**Authors:** Yijun Shao, Harmony S. Cen, Anu Dhananjay, S. J. Pawan, Xiaomeng Lei, Inderbir S. Gill, Anishka D'souza, Vinay A. Duddalwar
**Corresponding Author:** Yijun Shao (yijunsha@usc.edu)
**Affiliation:** Keck School of Medicine, University of Southern California, Los Angeles, CA, USA

**Online Resource 5.** Random Forest (RF) Performance Stratified by Grade Based on All Radiomic Features

| Biomarker | Grade 1/2 | | Grade 3/4 | | Difference | |
|---|---|---|---|---|---|---|
| | Correlation Coefficient (95%CI) | p value | Correlation Coefficient (95%CI) | p value | Correlation Coefficient (95%CI) | p value |
| **ANKZF1** | -0.1 (-0.34, 0.15) | 0.44 | -0.03 (-0.2. 0.15) | 0.78 | 0.07 (-0.23, 0.37) | 0.64 |
| **BCL2** | -0.03 (-0.27, 0.2) | 0.78 | 0.07 (-0.11, 0.24) | 0.46 | 0.1 (-0.2, 0.4) | 0.51 |
| **ETS1** | 0.14 (-0.06, 0.33) | 0.19 | 0.04 (-0.16, 0.24) | 0.7 | -0.1 (-0.38, 0.18) | 0.5 |
| **FBP1** | 0.11 (-0.25, 0.48) | 0.55 | -0.01 (-0.16, 0.15) | 0.91 | -0.12 (-0.52, 0.27) | 0.55 |
| **KLF6** | 0.04 (-0.15, 0.23) | 0.67 | 0.28 (0.07, 0.5) | <0.01* | 0.24 (-0.04, 0.53) | 0.1 |
| **PCK1** | -0.03 (-0.28, 0.21) | 0.79 | 0.08 (-0.1, 0.25) | 0.38 | 0.11 (-0.19, 0.42) | 0.47 |
| **PDK1** | -0.03 (-0.24, 0.18) | 0.8 | 0.09 (-0.11, 0.28) | 0.39 | 0.11 (-0.17, 0.4) | 0.44 |
| **PLAUR** | -0.15 (-0.51, 0.21) | 0.41 | 0.02 (-0.13, 0.17) | 0.8 | 0.17 (-0.22, 0.56) | 0.39 |
| **PLOD2** | -0.13 (-0.41, 0.14) | 0.34 | -0.07 (-0.24, 0.1) | 0.41 | 0.06 (-0.26, 0.38) | 0.7 |
| **PPARGC1A** | -0.12 (-0.38, 0.13) | 0.35 | -0.08 (-0.25, 0.09) | 0.38 | 0.05 (-0.26, 0.36) | 0.76 |
| **RORA** | -0.09 (-0.28, 0.1) | 0.36 | 0.2 (-0.01, 0.4) | 0.06 | 0.29 (0.01, 0.56) | 0.05* |
| **TEK** | -0.12 (-0.34, 0.1) | 0.28 | -0.12 (-0.3, 0.05) | 0.17 | 0 (-0.29, 0.28) | 0.98 |
| **WSB1** | -0.18 (-0.43, 0.07) | 0.16 | 0 (-0.18, 0.17) | 0.96 | 0.17 (-0.13, 0.48) | 0.26 |

* $p < 0.05$ indicates statistical significance.
