# Supplementary material for: Radiogenomic correlation of hypoxia-related biomarkers in clear cell renal cell carcinoma
Source: J Cancer Res Clin Oncol. 2025 Jun 12;151(6):186. doi: 10.1007/s00432-025-06240-8 (PMC12159112; doi:10.1007/s00432-025-06240-8)
Supplement: Supplementary file 6 — Supplementary Material 6 [file 432_2025_6240_MOESM6_ESM.pdf]

Article Title: Hypoxia-Related Gene Expression in Renal Cell Carcinoma
Journal Name: Clinical and Translational Oncology
Authors: Yijun Shao, Harmony S. Cen, Anu Dhananjay, S. J. Pawan, Xiaomeng Lei, Inderbir S. Gill, Anishka D'souza, Vinay A. Duddalwar
Corresponding Author: Yijun Shao (yijunsha@usc.edu)
Affiliation: Keck School of Medicine, University of Southern California, Los Angeles, CA, USA

Online Resource 6. Random Forest (RF) Performance Stratified by Stage Based on All Radiomic Features

Biomarker	Stage I/II		Stage III/IV		Difference	
	Correlation Coefficient (95%CI)	p value	Correlation Coefficient (95%CI)	p value	Correlation Coefficient (95%CI)	p value
**ANKZF1**	-0.03 (-0.22, 0.16)	0.78	-0.04 (-0.25, 0.17)	0.72	-0.01 (-0.3, 0.27)	0.94
**BCL2**	0.01 (-0.17, 0.19)	0.9	0.07 (-0.17, 0.3)	0.57	0.06 (-0.24, 0.35)	0.71
**ETS1**	0.13 (-0.03, 0.29)	0.12	-0.01 (-0.26, 0.24)	0.96	-0.13 (-0.43, 0.16)	0.38
**FBP1**	0.06 (-0.12, 0.24)	0.54	0 (-0.23, 0.24)	0.99	-0.05 (-0.35, 0.24)	0.72
**KLF6**	0.13 (-0.04, 0.29)	0.13	0.1 (-0.17, 0.37)	0.48	-0.03 (-0.34, 0.29)	0.87
**PCK1**	0.06 (-0.1, 0.21)	0.47	0.03 (-0.34, 0.4)	0.88	-0.03 (-0.43, 0.37)	0.89
**PDK1**	-0.03 (-0.2, 0.13)	0.69	0.07 (-0.18, 0.32)	0.58	0.1 (-0.2, 0.4)	0.5
**PLAUR**	0.06 (-0.24, 0.36)	0.71	0.01 (-0.14, 0.16)	0.9	-0.05 (-0.38, 0.29)	0.79
**PLOD2**	-0.07 (-0.28, 0.15)	0.53	-0.09 (-0.28, 0.1)	0.34	-0.02 (-0.3, 0.26)	0.88
**PPARGC1A**	-0.16 (-0.32, 0.01)	0.06	-0.03 (-0.31, 0.26)	0.86	0.13 (-0.2, 0.46)	0.43
**RORA**	0 (-0.15, 0.16)	0.96	0.1 (-0.21, 0.4)	0.54	0.09 (-0.25, 0.43)	0.6
**TEK**	-0.07 (-0.25, 0.1)	0.39	-0.13 (-0.38, 0.11)	0.28	-0.06 (-0.36, 0.24)	0.7
**WSB1**	-0.12 (-0.29, 0.05)	0.17	-0.12 (-0.36, 0.13)	0.36	0 (-0.3, 0.3)	0.99
