# Supplementary material for: Radiogenomic correlation of hypoxia-related biomarkers in clear cell renal cell carcinoma
Source: J Cancer Res Clin Oncol. 2025 Jun 12;151(6):186. doi: 10.1007/s00432-025-06240-8 (PMC12159112; doi:10.1007/s00432-025-06240-8)
Supplement: Supplementary file 7 — Supplementary Material 7 [file 432_2025_6240_MOESM7_ESM.pdf]

Article Title: Hypoxia-Related Gene Expression in Renal Cell Carcinoma
Journal Name: Clinical and Translational Oncology
Authors: Yijun Shao, Harmony S. Cen, Anu Dhananjay, S. J. Pawan, Xiaomeng Lei, Inderbir S. Gill, Anishka D'souza, Vinay A. Duddalwar
Corresponding Author: Yijun Shao (yijunsha@usc.edu)
Affiliation: Keck School of Medicine, University of Southern California, Los Angeles, CA, USA

Online Resource 7. Top 3 most predictive radiomic features by gene ranked in descending order. Naming convention generally follows <family>_<plane>_<phase>_<metric>. See Table 4 and Online Resource 8 for definitions. Common features identified across multiple genes have been bolded for emphasis.

Biomarker	Top 3 Features
**ANKZF1**	GC2_Cor_ART_Uniformity
	GC3_VEN_CON
	LE2_Sag_VEN_L5W5
**BCL2**	**GD2_Cor_DEL_Uniformity**
	LR2_Sag_PRE_LGRE
	GD2_Axi_DEL_difENT
**ETS1**	GC3_VEN_ASM
	GC3_VEN_Uniformity
	NT2_Sag_PRE_TexStren
**FBP1**	DT2_Axi_DEL_var_vB_4
	GC2_Cor_DEL_MCC
	LS2_Axi_ART_ZP
**KLF6**	GD2_Axi_VEN_HOM
	LR3_DEL_RP
	DT2_Axi_DEL_skew_dB_4
**PCK1**	GC2_Axi_ART_SQV
	GC2_Axi_ART_STD
	DT2_Cor_PRE_skew_dB_4
**PDK1**	DT2_Cor_VEN_skew_vB_2
	GD2_Sag_PRE_Corr
	INT_DEL_Min
**PLAUR**	DT2_Sag_VEN_kurt_2dB_1
	DT2_Sag_VEN_kurt_dB_1
	DT2_Sag_VEN_kurt_vB_1
**PLOD2**	GC2_Axi_ART_difAve
	GD2_Cor_VEN_Corr
	INT_DEL_STD
**PPARGC1A**	LR3_DEL_HGRE
	LR3_DEL_LRHGE
	NT2_Sag_VEN_Busy
**RORA**	LS2_Sag_PRE_Nz
	**GD2_Cor_DEL_Uniformity**
	GD2_Sag_PRE_ENT
**TEK**	LE3_PRE_L5L5E5
	DT2_Axi_ART_mean_vB_1
	DT2_Axi_PRE_skew_hB_2
**WSB1**	DT2_Cor_ART_mean_vB_1
	DT2_Axi_ART_mean_hB_3
	GC2_Axi_ART_DIS
