# Supplementary material for: Radiogenomic correlation of hypoxia-related biomarkers in clear cell renal cell carcinoma
Source: J Cancer Res Clin Oncol. 2025 Jun 12;151(6):186. doi: 10.1007/s00432-025-06240-8 (PMC12159112; doi:10.1007/s00432-025-06240-8)
Supplement: Supplementary file 8 — Supplementary Material 8 [file 432_2025_6240_MOESM8_ESM.pdf]

Article Title: Hypoxia-Related Gene Expression in Renal Cell Carcinoma
Journal Name: Clinical and Translational Oncology
Authors: Yijun Shao, Harmony S. Cen, Anu Dhananjay, S. J. Pawan, Xiaomeng Lei, Inderbir S. Gill, Anishka D'souza, Vinay A. Duddalwar
Corresponding Author: Yijun Shao (yijunsha@usc.edu)
Affiliation: Keck School of Medicine, University of Southern California, Los Angeles, CA, USA

Online Resource 8. Radiomic Feature Abbreviations and Definitions. Summary of anatomical planes, contrast-enhanced imaging phases, and quantitative texture metrics used in radiomic feature extraction for hypoxia-related biomarker modeling.

Feature Type	Abbreviation	Definition
**Anatomical Plane**	Axi	Axial, Horizontal slice
	Cor	Coronal, Vertical slice (front-to-back)
	Sag	Sagittal, Vertical slice (side-to-side)
**Imaging Phase**	PRE	Pre-contrast, before contrast injection
	ART	Arterial, early post-contrast
	VEN	Venous, later post-contrast
	DEL	Delayed, much later post-contrast
**Texture Metric**	mean	Average voxel intensity
	var	Variance of voxel intensities
	std	Standard deviation of voxel intensities
	skew	Asymmetry of intensity distribution
	kurt	Kurtosis - Peakedness (tailedness) of intensity distribution
	Min	Minimum voxel intensity
	SQV	Sum of Squared Variance (total variability of texture intensities)
	ASM	Angular Second Moment (texture uniformity)
	ENT	Entropy (texture randomness)
	DIS	Dissimilarity (intensity difference between neighbors)
	Corr	Correlation (linear relationship between neighbors)
	MCC	Maximal Correlation Coefficient (spatial complexity)
	difENT	Difference Entropy (entropy of pixel differences)
	Uniformity	Uniformity across pixel intensities
	CON	Contrast between neighboring voxels
	HOM	Homogeneity (local similarity of intensities)
	RP	Run Percentage (density of intensity runs)
	LGRE	Low Gray Level Run Emphasis (long runs of low intensities)
	HGRE	High Gray Level Run Emphasis (long runs of high intensities)
	LRHGE	Low Run High Gray Level Emphasis (short runs of high intensities)
	ZP	Zone Percentage (zones relative to total region)
	Nz	Non-zero Zones (count of meaningful zones)
	Busy	Busyness (rate of local intensity change, visual clutter)
	TexStren	Texture Strength (overall strength of perceptible texture patterns)
	L5L5E5 and L5W5	Laws Texture Energy (different fine texture filters combinations)

dB = diagonal band, vB = vertical band, hB = horizontal band; refers to directional sub-bands in the 2D Discrete Cosine Transform (DCT) decomposition.
